# The prognostic value of perifollicular blood flow in the outcome after assisted reproduction: a systematic review

**Published:** 2017-09

**Authors:** S Huyghe, A Verest, A Thijssen, W Ombelet

**Affiliations:** Genk Institute for Fertility Technology, Department of Obstetrics and Gynaecology, ZiekenhuisOost-Limburg, Schiepse Bos 6, 3600 Genk, Belgium; Faculty of Medicine, KULeuven, Gasthuisberg, Herestraat 49, 3000 Leuven, Belgium; Department of Gynecology and Obstetrics, Ziekenhuis Oost-Limburg, Schiepse Bos 6, 3600 Genk, Belgium; Faculty of Medicine and Life Sciences, Hasselt University, Martelarenlaan 42, 3500 Hasselt, Belgium.

**Keywords:** assisted reproduction technologies, ICSI, IUI, IVF, perifollicular blood flow, prognostic marker, fertilization rate, pregnancy rate

## Abstract

**Background:**

The overall final outcome of assisted reproductive technologies (ART) is still more often a failure than a success. Assessing perifollicular blood flow (PFBF) is one technique to predict and possibly improve this outcome. The aim was to provide a structured review of studies concerning PFBF and its prognostic value in patients undergoing ART, including IUI (intrauterine insemination).

**Methods:**

PUBMED, EMBASE and Cochrane Database of Systematic Reviews were searched for relevant studies published until December 2016. As key words ‘Perifollicular blood flow’, ‘IUI’, ‘IVF’ and ‘ICSI’ were used.

**Results:**

A total of 14 articles were included in the current review. The results are very heterogeneous, though there is evidence that measuring PFBF could be a good prognostic marker for oocyte and embryo quality, but even more for pregnancy rate after IVF/ICSI. This finding is not observed in studies concerning IUI.

**Conclusions:**

Our results highlight an urgent need to investigate the role for PFBF assessment by Power Doppler in ART in randomised controlled trials.

## Introduction

An estimated 48,5 million couples worldwide suffer from infertility (Mascarenhas et al., 2012). Different causes for infertility or subfertility are known e.g. ovulatory disorders, decreased ovarian reserve, tubal blockage, uterine factors, endometriosis and decreased sperm quality. For subfertile couples there are different treatment pathways, depending on the reason and duration of subfertility. The most used modalities in assisted reproduction treatments (ART) worldwide are Intrauterine insemination (IUI), In vitro fertilization (IVF) and Intracytoplasmic sperm injection (ICSI).

ART is still a relatively new field in medicine, with the first IVF baby born in 1978 (Steptoe and Edwards, 1978). The overall final outcome of ART is still more often a failure than a success. According to the Belgian registration a clinical pregnancy rate (CPR) of 25,5 % per aspiration and 31% per transfer could be observed after IVF/ICSI (BELRAP, 2014). The 2012 European registry by EHSRE showed a CPR of 29,4% and of 33,8% per aspiration and per transfer respectively V for IVF. For ICSI the corresponding rates were 27,8% and 32,3% (Calhaz-Jorge et al., 2016).

Different steps have been examined to improve the still rather low success rate after ART. Improvements are recently made for example by performing ultrasound guided embryo transfers (Buckett, 2003) and blastocyst transfers (Glujovsky, 2016). Clinicians are keen to try new ideas/ techniques especially after failed attempts. It is of utmost importance to keep the balance between costs, possible harm done and actual advantage.

The recognition and selection of high-quality oocytes is a key factor in the success of ART. In this review perifollicular blood flow (PFBF) is studied as a predictor for good quality of follicles, oocytes and embryos, as well as for pregnancy outcome after ART. Folliculogenesis defines the progress of a primordial follicle to a mature follicle. It is a complicated process, which includes dynamic and endocrine changes. The ripening of the follicle results in a capillary network that mediates the transport of oxygen, nutrients and precursor-substances. Vascularization is the primary essential step in follicular growth and the follicular microenvironment is an essential factor in oocyte growth (Vural et al., 2016). In this way it seems understandable that folliculogenesis can be a process of interest to improve results of ART. A lot of prognostic markers have been demonstrated to contribute to evaluate the quality of follicles such as increased proliferation, size of the follicle, amount of vascular endothelial growth factor (VEGF) in follicular fluid and PFBF. Assessment of PFBF provides a safe, inexpensive and easy way to evaluate the blood flow around the follicle, though it is still a somewhat controversial method that is not widely incorporated in IVF trials, hence this systematic review for its value and predictive power.

### Assessment of the PFBF

To demonstrate the value of this indicator, we need to standardise its measurement. This can be done in either a qualitative or a quantitative way, both by a single transvaginal Doppler ultrasound scan on the day of hCG administration or just before ovarian pick-up procedure. Mostly used is the qualitative way in which Power Doppler ultrasound is applied to assess and describe the vascularity of the follicles. Compared to colour Doppler imaging, power Doppler ultrasound has a superior visualisation of small vessels and it has a higher sensitivity for detecting low velocity flow (Borini et al., 2004). The study by Chui et al. (1997) is frequently used as classification regarding qualitative assessment of PFBF (Figure I, Table 1).

**Figure 1:**
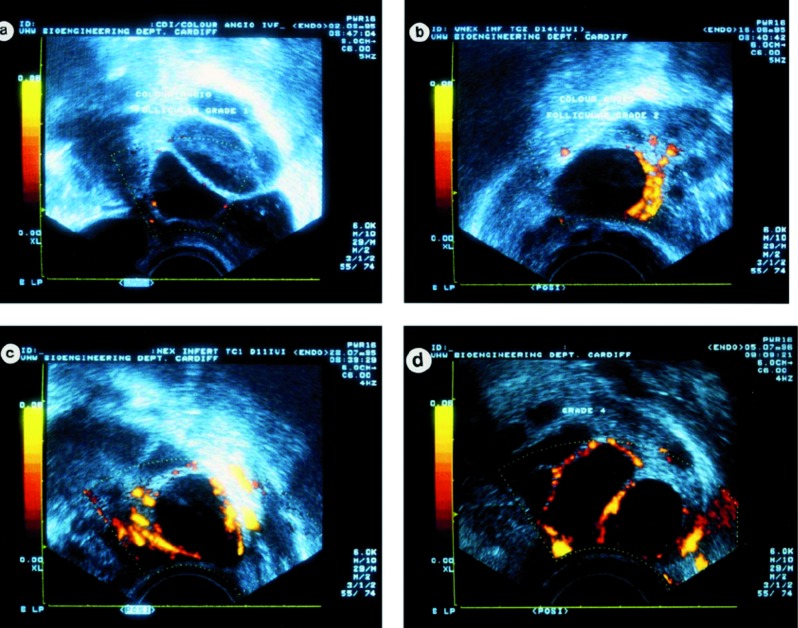
— Power Doppler scans showing grading system used to assess follicular vascularity. (a) Shows 25% circumferential flow (grade F1); (b) 26–50% flow (grade F2); (c) 51–75% flow (grade F3) and (d) >75% flow (grade F4). (Chui et al., 1997)

**Table I:** Grading system for the follicles vascularity, expressed in percentage of follicular circumference in which flow was identified. (Chui et al., 1997)

F1	<25% of the follicular circumference
F2	25-50% of the follicular circumference
F3	51-75% of the follicular circumference
F4	>75% of the follicular circumference

## Materials and methods

### Search strategy

The goal of this structured review was to investigate the predictive value of the perifollicular flow on the outcome of assisted reproduction. The keyword used was: ‘perifollicular blood flow’. The databases used were Pubmed from 1966, Cochrane library from 1993 and EMbase from 1974 until 2016. This search gave 52 hits on Pubmed and 66 hits on EMbase, none in the Cochrane Database. When these hits were checked for similarity, we included 79 different publications. Secondly `IVF ´, `IUI ´ and `ICSI ´ were used as combination key words. A total of 43 different articles (IVF 25, IUI 10, ICSI 8 publications) could be selected in both databases.

### Criteria for considering studies

**Figure 2:**
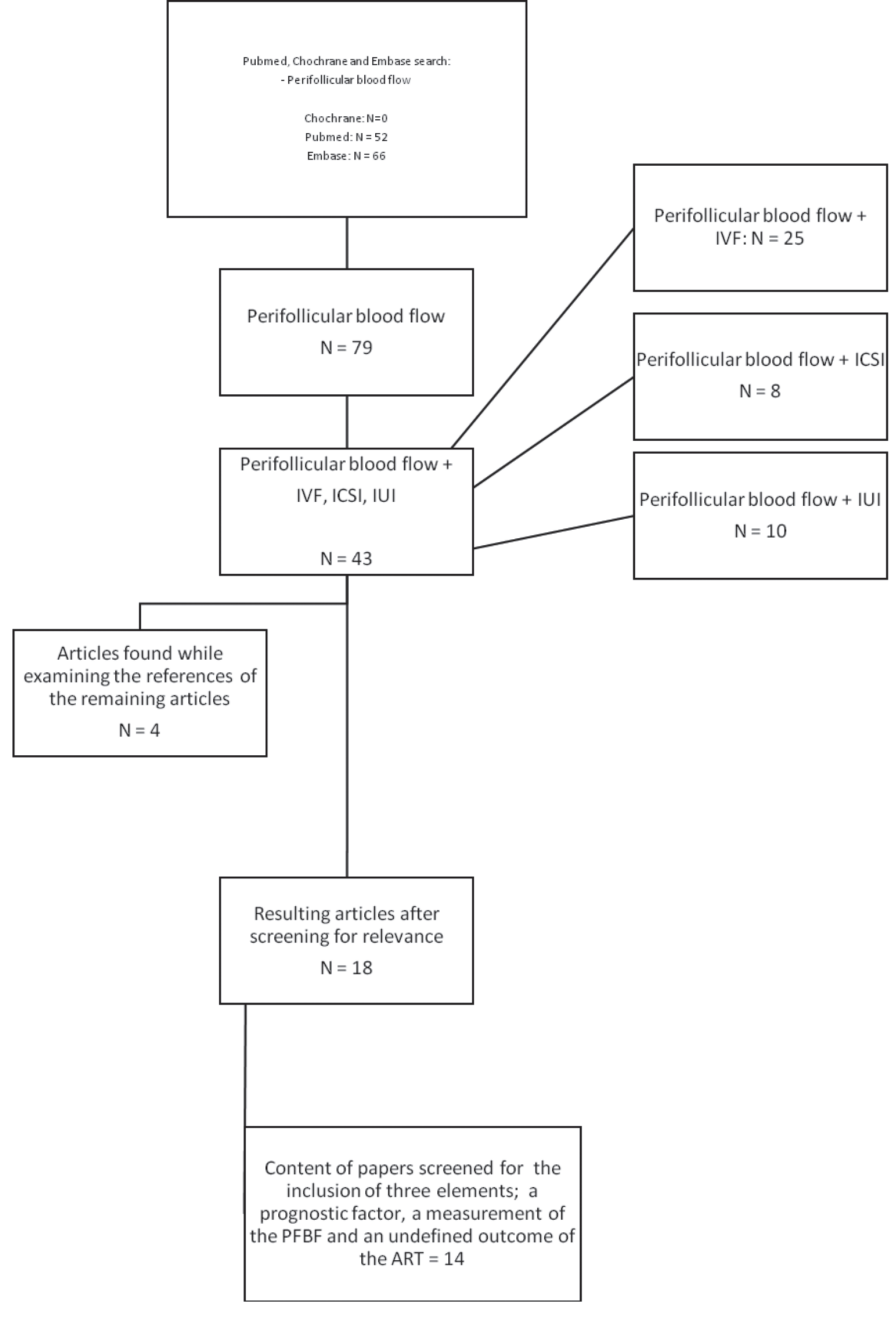
— Overview of the search strategy

These 43 articles were thereafter screened on the content and relevance of the title and abstract by two readers, S.H. and A.V. The articles that focused on the outcome of assisted reproduction with PFBF as the main predictor were included. The studies which had their main focus on the technique of assisted reproduction, studies with non-human subjects or studies that determined which method of ultrasonography is best to use were excluded. Studies focusing on a specific clinical situation, like genital tuberculosis, were also excluded. The 14 eligible articles were then checked for the inclusion of three elements; a prognostic factor, a measurement of the PFBF and an undefined outcome after ART treatment. 10 articles remained. Whilst examining the references of these studies, four more were included, which leaves us at a total of 14 articles, 12 on IVF/ICSI and 2 on IUI. An overview of the search strategy is shown in Figure 2.

### Review method

In this systematic review the 14 articles are compared in an attempt to reach a general conclusion about influence of PFBF on outcome of ART, despite their differences in ways of measuring PFBF and outcome parameters. The articles concerning IVF and ICSI will be reviewed chronologically in time, and afterwards the two studies regarding IUI.

## Results

The 14 selected studies are different in patient selection and outcome parameters. Most studies investigate the pregnancy rate as outcome. Others, like Huey et al. (1999) measure the fertilisation and day 3 embryo cleavage rate. Another inter- study difference is the qualitative or quantitative measurement of the PFBF. An overview of the results is given in Table II.

**Table II:** Overview of the results, chronologically, 12 articles concerning IVF and 2 on IUI.

Author	Year	N° of patients	Age	ART	Oocyte retrieval rate	Oocyte quality	Fertilization rate	Embryo quality	Pregnancy rate
Nargund et al.	1996	27	29-43	IVF	Positively correlated with PFBF	NA	NA	Postively correlated with PFBF	NA
Huey et al.	1999	16	21-39	IVF	NA	NA	Negatively correlated with RI and S-D ratio.	Embryocleavage is negatively correlated with RI, PI and S-D ratio	NA
Coulam, Goodman and Rinehart	1999	106	21-37	IVF	NA	NA	NA	NA	Positively correlated with PFBF and PSV
Bhal et al.	1999	179	31-35	IVF	Positively correlated with PFBF	MFD is positively correlated with PFBF	Positively correlated with PFBF	Embryo morphology is independent of PFBF, less triploid embryos	Positively correlated with PFV
Kim et al.	2004	47	34±43	IVF	NA	Size of the follicles is correlated with PFBF	NA	NA	Positively correlated with PFV
Borini et al.	2004	43	30-38	IVF	NA	NA	NA	NA	Significantly higher pregnancy rate in the group with high vascularization in comparison with the mixed group
Palomba et al.	2006	54	18-30	IVF	No difference between the 2 groups	No difference between the 2 groups. Association with good morphology.	No difference between the 2 groups	No difference between the 2 groups	No difference between the 2 groups
Palomba et al.	2008	60	>40	IVF	No significant difference between the 2 groups	No significant difference between the two groups	Significantly higher in the FSH < 10,9 IU/L population	Embryo cleavage is significantly higher in the FSH <10,9 IU/L population	Significantly higer in the FSH <10,9 IU/L population
Monteleone et al.	2008	61	<35	IVF / ICS	NA	NA	NA	Positively correlated with PFBF	Positively correlated with PFBF
Robson et al.	2008	26	NA	IVF / ICS	No significant difference between the 2 groups	NA	No significant difference between the 2 groups	NA	No statistically relevant correlation with PFBF (positive trend)
Vural et al.	2016	40	23-35	IVF / ICS	NA	NA	NA	Positively correlated with PFBF	Positively correlated with PFBF
Ragni et al.	2007	318	30-40	IUI	NA	NA	NA	NA	Independent of PFBF
Panchal and Nagori	2009	1000 cycli	NA	IUI	NA	NA	NA	Positively correlated with PFBF	Positively correlated with PFBF

(IUI = intrauterine insemination, IVF = in vitro fertilization, ICSI = intracytoplasmatic Sperm injection, NA = not applicable, PFBF = perifollicular blood flow, PSV = peak systolic velocity, FSH = follicle stimulating hormone, MFD = Mean follicle diameter, RI = resistance index, PI = pulsatility index, S-D = Systolic-diasdiastolic, IU = international units)

Nargund et al. (1996) performed a prospective study to investigate the link between follicular blood flow, oocyte retrieval and pre-implantation embryo quality. They included 126 follicles in 27 women treated with IVF. Parameters were follicular volume, perifollicular blood flow with calculation of PI and PSV, oocyte retrieval and morphological grade of each pre-implantation embryo. A significant difference was found in oocyte retrieval when PFBF was considered (P<0.05). The follicles with detected PFBF (PSV ≥3 cm/s) contained significant more oocytes. Also a borderline significant difference was seen in the quality of the pre-implantation embryos. Follicle size was not associated with number of oocytes retrieved nor with their quality.

In a prospective study with 38 patients undergoing IVF treatment Chui et al. (1997) investigated PFBF qualitatively and they installed a grading system. The vascularity of the follicles was graded with respect to the percentage of follicular circumference in which flow was identified from a single cross- sectional slice. As mentioned before, the grading system subdivided the follicles into 4 groups (Table I). The results of this study demonstrated that the follicular size at the moment of oocyte retrieval rate was independent of the vascularity grading but that there was a trend of higher but not significant fertilisation potential with increasing vascularity grade. Furthermore, a significantly higher proportion of triploid embryos were observed with oocytes that derived from follicles with poor vascularisation (25-30% for F1 and F2 follicles), than from those with higher vascularisation (<6% for F3 and F4 follicles). Pregnancies were only seen in those women whose embryos were derived from follicles with grade 3 and 4 vascularity (pregnancy rates per ET of respectively 12,5% versus 61,5%) with only those from grade 4 follicles resulting in livebirths.

Huey et al. (1999) conducted a prospective study with 16 women to determine the predictors for embryo quality and fertilisation. Among the investigated predictors were the Doppler blood flow indices PI, RI, the peak flow velocity (PVmax) and the S-D ratio (a simpler index of the pulsatility). On the day of the aspiration, the follicles of these women were mapped in longitudinal and transversal planes, placed on a hard copy, used by the surgeon to retrieve the studied oocytes. Only three follicles were studied per ovary because of the possible changes in the ovarian architecture with each aspiration. Oocyte insemination was performed in a regular IVF or through ICSI. Day three transfers were performed. The fertilisation rate was 69.8%. The results of a correlation analysis showed that all Doppler indices except PVmax (RI, PI and S-D) were significantly and negatively correlated with embryo cleavage. No correlation existed between the Doppler indices and embryo morphology. As no difference was made between study follicles and non-study follicles during the transfer of the embryos, relationships with implantation rates could not be evaluated. ROC analysis was performed on the Doppler indices PI, RI and S-D to determine the predictive value on a good quality embryo (≥8 blastomeres on day 3) and showed an area under the curve of 0.65, 0.64 and 0.65 for RI, PI and S-D respectively. The blood flow indices are therefore moderate predictors of embryo cleavage.

Coulam et al. (1999) investigated the value of quantitative and qualitative indices of follicular vascularity (PFBF) in predicting pregnancy rates on a prospective basis. They included 106 women with a high risk for failure of IVF (>37 years, low responder in earlier cycles and/or failed IVF cycles in the past). The three largest follicles per ovary were included. For qualitative measurement of PFBF they used the gradation like Chui et al. (1997) (Table I), for quantitative measurement PSV was used. They analysed 565 follicles, the best results were seen in the group with PSV ≥ 10cm/s and PFBF grade III-IV (p=0.05).

Bhal et al. (1999) performed a larger study of 200 treatment cycles to assess the subjectivity of the vascularity grading system used by Chui et al (1997) (Tabel I). Vascularity was assessed by an ultrasound scan, performed on the day of oocyte retrieval. A total of 1285 follicles were studied. The grade 1 and 2 follicles were considered to be of low grade vascularity and the grades 3 and 4 were considered to be of high grade vascularity. Mean follicular diameter (MFD) was measured and observed to increase significantly as the vascularity grade increased. Additionally the oocyte retrieval, maturity and fertilisation rates were found to be higher in the high grade group, and the triploidy rates perceived to be lower. To assess the clinical pregnancy rates, the cycles were split into three groups; one group contained only embryos derived from high grade follicles, one with embryos exclusively of a low-vascular origin and a mixed group. Based on this analysis, an increased pregnancy rate and a decreased pregnancy loss were demonstrated in treatment cycles with embryos from follicles with uniformly high vascular grading.

Another prospective study performed by Kim et al. (2004) investigated the relationship between a quantitative measurement of PFBF, with PI as index, and the pregnancy outcome after IVF. 47 cycles of IVF were executed with controlled ovarian hyperstimulation using the GnRH agonist buserelin acetate. In this study, the follicles were examined with an ovarian ultrasound on the day of hCG administration, 34-35 hours before oocyte retrieval. The average amount of embryos transferred was 4.3 ±1.7. Of the 47 cycles, 38.3% resulted in pregnancy with the blood flow being significantly higher (lower PI) in the pregnant group compared with the non-pregnant group (p<0.05). There was also a significant positive correlation found between the PFBF and the size of the follicle.

In 2004 Borini et al. (2004) analysed the relationship between perifollicular (PF) vascularity and the outcome of IVF cycles within a group of 43 patients with a mean age of 34 years. The total numbers of stimulated follicles ranged from 6 to 16. Couples with severe male factor infertility and women who did have more than one previous failed cycle were excluded in this study. The vascularity grades were the same as those from Chui et al. (1997) (Table I) and the subjects were divided in 3 groups. Group 1 had PF vascularity grades 3-4, group 2 had FV grades 1-2 and group 3 had mixed FV grades (control group). This study showed pregnancy rates of 55%, 33.3% and 15% in group 1, 2 and 3 respectively: statistically higher pregnancy rate in the group with high vascularisation in comparison with the control group.

Palomba et al. (2006) conducted a pilot study after the constitution of a new law in Italy, imposing a ban on the fertilisation of more than 3 oocytes at a time. They enrolled 54 primary infertile non obese women with an age ranging from 18-30. The subjects were equally subdivided in an experimental and a control group (27/27). In the experimental group, on the day of hCG injection and the day of oocyte retrieval, a power Doppler assessment was made of the PF vascularity, which was graded analogous to the vascularity grading in Chui et al (1997) (Table I). In the experimental group both morphological criteria and PF vascularisation were used to select oocytes. In the control group, however, the morphological criteria alone were used for selection of the best 3 oocytes to fertilise. The two groups were similar in implantation and no significant difference in pregnancy rate existed. The PF vascularity however, turned out to be a good predictor of oocyte quality. In particular 77.0%, 28.3% and 5.0% of the grade I (best morphology grade), II and III (worst morphology grade) oocytes, respectively, showed a high grade PF vascularity. These results corresponded with a sensitivity of 68.1% and a specificity of 93.2% for the PF vascularity in the detection of a grade I oocyte morphology score.

In a second study Palomba et al. (2008) examined the PF vascularity assessment for selecting the best oocytes in 60 patients who were > 40 years of age. They subdivided the 60 patients again into 2 groups: the experimental group and the control group. Similarly the top 3 oocytes in the control group were chosen based only on their morphological grading. The 3 chosen oocytes in the experimental group were again dependent on both the morphological and the PF vascularity grading. They concluded that FSH was the main factor influencing ongoing pregnancy rate, a good ovarian reserve is needed to even have enough follicles to select from.

Monteleone et al. (2008) investigated the correlation between perifollicular blood flow and vascular endothelial growth factor (VEGF) prospectively. 61 Consecutive patients were included, all during their first IVF cycle. For all patients infertility was due to male factor or tubal factor. At oocyte retrieval the perifollicular vascularity was estimated by power Doppler blood flow and classified by the classification of Chui et al (1997) (Table I) for a total of 244 follicles. The follicular fluid was centrifuged and stored until VEGF assay. They found VEGF levels to be significantly correlated with grade of perifollicular vascularity. They also showed a significantly higher percentage of grade A embryos when oocytes deriving from highly vascularized follicles were used and the pregnancy rate per embryo transfer in women with embryos deriving from follicles with a high grade of vascularization (F3/F4) was significantly higher (21.1% in F1/F2 follicles versus 37.5% in F3/F4 follicles). The investigators argue that follicles with high grade of vascularization need to be selected for better embryo quality.

Robson et al. (2008) assessed the practicality of using power Doppler to determine the PF vascularity at the time of oocyte retrieval. 26 Women undergoing IVF treatment were recruited and a total of 275 follicles were studied. No difference was found in oocyte yielding or fertilisation rates between the low- and high-grade follicles. Because the embryo transfer cohorts were mixed it was not possible to calculate the specific implantation rates. The mean numbers of embryos transferred were 2.14 in the pregnant group and 2.38 in the non-pregnant group. When at least one of the embryos transferred was from a grade 3 or 4 follicle, the clinical pregnancy rate was 50 % in contrast with a rate of 15.4% when none of the embryos was derived from a grade 3 or 4 follicle. This corresponds with a P-value of 0.74, probably due to the small sample size.

Vural et al. (2016) investigated the association of PFBF with follicular fluid (FF) EG-VEGF, inhibin-a, IGF-1 concentrations, endometrial vascularity and finally IVF outcomes in a prospective cohort study including 40 women with tubal infertility. A total of 156 follicles were assessed by power Doppler ultrasonography and classified as well- vascularised ((WVF), 51-100% PF vascularity) or poorly vascularised (PVF). Briefly, this study demonstrated that WVF’s yielded high percentages of good-quality embryos, a well vascularised endometrium, high FF EG-VEGF levels and high clinical pregnancy rates. In addition, pregnant women had an increased percentage of WVF’s, a well vascularised endometrium and elevated FF IGF-1 and serum EG-VEGF levels but similar FF EG-VEGF and serum inhibin-a levels. They suggested an essential role for Doppler assessments of PFBF and endometrial blood flow in all IVF/ICSI procedures. PFBF and FF IGF-1 and serum EG- VEGF might be independent markers for pregnancy outcomes.

We also included two studies who investigated PFBF as a prognostic marker in an IUI-program.

Regarding the predictive value of the PF vascularity in IUI, Ragni et al. (2007) conducted a study with a total of 318 women, less than 40 years of age, undergoing mild controlled ovarian stimulation. The PF vascularity was assessed in all follicles with a mean diameter ≥16mm and were either said to be low-vascularised (follicular circumference in which flow is identified <1/3), medium-vascularised (1/3< follicular circumference with identified blood flow <2/3) or high-vascularised (>2/3). When more than one follicle was graded within a subject, the PF vascularisation grade of the follicle with the best blood flow was assigned to be representative. In total there were 38 pregnancies without a significant difference between the clinical pregnancy rates in the low-, medium- and high-grade vascularity groups, which were 14.1%, 10.0% and 11.8% respectively.

Another study concerning perifollicular blood flow as a predictor in IUI by Panchal and Nagori (2009) looked at 1000 IUI cycles and assessed the blood flow quantitatively at the time of the hCG injection. They concluded that a perifollicular RI (Resistance Index) <0.50 and PSV (Peak systolic velocity) > 11 cm/s was more favourable for conception than RI > 0.50 and PSV <11 cm/s (conception rates RI: 32.3 and 10.76% respectively, conception rates PSV: 27.0 and 14.2% respectively). This study is the only one looking into the volume and the global vascularity of the follicle by using a 3D vocal system. Even when the follicle appeared mature according to the 2D ultrasound (US), the pregnancy rates were significantly better only when the follicular volume was between 3 and 7cc (cubic centimeters), cumulus was present, the perifollicular vascularity index (VI, calculated flow in the whole volume of the follicle) was between 6 and 20 and the flow index (FI, calculated intensity of flow in the volume of the follicle) more than 27.

## Discussion

### Principal findings

The past two decades a considerable amount of factors predicting the outcome of ART have been suggested. One of them is PFBF. In this systematic review, a literature search was performed to analyse the outcomes of various studies concerning this topic and to reach an acceptable conclusion. There is definitely a positive trend towards predicting outcome of ART, more clearly seen in IVF than in IUI. Due to the heterogeneity of the reviewed studies a firm conclusion is not possible.

### Strengths and weaknesses

This is the first systematic review on this subject. An extensive literature research was conducted. The publications considered in this review were all prospectively carried out, though rather poorly sized and an inter-study difference existed in the procedures used to perform IVF and to asses PFBF, either qualitatively or quantitatively. Due to the small sample sizes the sometimes contradicting results may not be of much value. Moreover not all studies have focused on the same outcomes. This makes it impossible to pool the data and to draw a general conclusion.

### Interpretation according to outcome

For this reason we have discussed the selected papers per outcome measure. The outcome of ART can be defined in many ways. Here, we have focused on 5 of them: oocyte retrieval rate, oocyte quality, fertilisation, embryo quality and the pregnancy rate.

Three studies stated that the follicular vascularity grading was independent of oocyte retrieval rate (Chui et al., 1997; Palomba et al., 2006; Robson et al., 2008). On the other hand Oyesanya et al. (1996) defined a follicular vascularity index (the proportion of follicles demonstrating pulsatile vascularity) which was higher in the groups with the highest recovery rate. Bhal et al. (1999) concluded that oocyte retrieval rate was significantly higher when high and low PF vascularity grades were compared. Nargund et al. (1996) found a significant relationship (p<0.0001) between whether or not PFBF was detected and whether or not an oocyte was recovered. A test based upon the presence of detectable blood flow for the subsequent recovery of an oocyte yielded a sensitivity of 74% and a positive predictive value of 93% (Nargund et al., 1996).

There was no difference in oocyte quality between the groups with high and low PF vascularity grades in the study of Palomba et al. (2006) however they did find an interfollicular significant correlation of the PF vascularity and the oocyte morphology. It is well known that oocytes are sensitive to hypoxic damage and that oocytes from severely hypoxic follicles are associated with high frequencies of abnormalities in the organisation of the chromosomes (Van Blerkom et al., 1997). A good oxygenation is essential for the development of the oocytes and the differences in dissolved oxygen content of the follicular fluid correlate well with the differences in corresponding PF vascularity (Van Blerkom, 1998). The statement that follicular size correlates positively with PF vascularity is supported by two studies, which reported that the mean follicular diameter increased marginally but significantly as the vascularity grade increased (Bhal et al., 1999; Kim et al., 2004). A possible explanation is that high grade vascularity follicles are more mature. The degree of PF vascularity pre-hCG may affect the uptake of this hormone and impaired uptake can lead to impaired maturation. Likewise, a higher grade perfusion may lead to increased access of FSH to the follicle, also promoting maturation. In a similar study Chui et al. (1997) observed an insignificant relationship between the two and found them to be independent.

Concerning fertilisation rate Robson et al. (2008) found no significant difference between the low- and high grade follicles. Neither did Chui et al. (1997) and Palomba et al. (2006). The study of 2008 with patients > 40 years, however, had an ambiguous outcome (Palomba et al., 2008). A significant difference in fertilisation rate was described between the experimental and the control group in the population sample with FSH serum levels <10.9 IU/L, whilst no significant discrepancy was found in the group with serum levels >10.9 IU/L. The same conclusion can be drawn for the embryo quality and the ongoing pregnancy rate. A corresponding positive correlation for the fertilisation rate and PF vascularity was reported by Bhal et al (1999). However Huey et al. (1999) showed a positive correlation between the fertilisation rate and the Doppler indices RI and S-D. Therefore no firm conclusions can be drawn of the prognostic value of PFBF on neither oocyte retrieval rate, oocyte quality nor fertilisation rate.

Nevertheless it is confirmed that PF Doppler analysis, performed at a late stage of controlled ovarian hyperstimulation, provides an indirect index of embryo development in IVF (Chui et al., 1997; Van Blerkom et al., 1997; Huey et al., 1999; Van Blerkom, 1998). And concerning the pregnancy rate, there is a congruent conclusion amongst all but one study; the pregnancy rate for cycles with transferred embryos derived from follicles with a high grade vascularity, is significantly higher than for the cycles with embryos transferred from mixed or low grade follicles (Chui et al., 1997; Bhal et al., 1999; Borini et al., 2004; Kim et al., 2004; Robson et al., 2008). There is only one study where no relationship was found (Palomba et al., 2006). This discrepancy could be explained by the age of the population sample in this particular study. The subjects were between 18 and 30 years of age whereas most other study samples contain patients of >30. It is possible that younger women have a higher number of oocytes of an optimal quality, proven by the strong association between optimal quality oocytes and high grade follicles in this study. The study with older patients concluded that the pregnancy rate was significantly different between the groups if a normal ovarian reserve is considered (Palomba et al., 2008).

Our structured review indicates that the literature on the role of PFBF on predicting outcome of IUI is very limited. The two selected studies were in contradiction of one another. Ragni et al. (2007) stated that PF vascularity did not have any predictive value while Panchal and Nagori (2009) found the PSV to be positively and the RI negatively related with the pregnancy rate in women undergoing IUI. Those last two authors add an assesment method by considering vascularity and flow indices calculated in 3D on the whole volume of the follicle suggesting 3D ultrasound and 3D power Doppler are even more accurate on PFBF assessment.

## Conclusion

ART results have improved significantly over the past 30 years. Nevertheless we are still searching for techniques to improve the success rates. The purpose of this review was to look into PFBF assessment as a possible technique to predict and optimize results of ART. Research of the literature surrounding this subject, demonstrated an important prognostic value of PF Doppler analysis on the pregnancy rate after IVF. This prognostic value is rather contradictory in IUI. PFBF also showed a positive correlation with embryo quality. An indirect influence of PFBF on the pregnancy rate is assumed through formation of a more mature follicle, a more resistant oocyte and a better embryo cleavage potential. Both quantitative, in 2D and 3D, and qualitative measurements of PFBF are used. All selected studies have rather small sample sizes, all differ in ART methods used and vary in defined outcome. Therefore this review lacks a firm conclusion and it highlights the need for further research by performing randomised controlled trials, taking into account the inexpensive and rather easy nature of the PFBF assessment. Such a randomised trial should choose one method of PFBF measurement, either quantitative or qualitative, a patient group with a normal ovarian reserve and a well-defined outcome eg. clinical pregnancy rate. Preferably this should be a multicentric study with randomisation between either PFBF assessment or not. Major constraint might be the time-consuming aspect of PFBF measurement.
